# Philosophical Transactions of the Royal Society of London, Vol. LXXIII. For the Year 1783. Part II

**Published:** 1784

**Authors:** 


### LONDON MEDICAL JOURNAL,

For JULY, AUGUST, and SEPTEMBER, 1784.

### SECTION I.

### BOOKS.

I. *Philosophical Transactions of the Royal Society of London, vol.* LXXIII. *for the year* 1783. *Part* II. 4to. London, 1784. 255 pages, with 7 copper plates.

1. *MEMOIRE sur la maniere de preparer, avec le moins de perte possible, le sel fusible d'Urine blanc, & pur, & l'Acide phosphorique parfaitement transparent*, i. e. *Essay on the manner of preparing, with the least possible loss, the fusible salt of urine white and pure, and the phosphoric acid perfectly transparent. By the Duke de* Chaulnes, F.R.S.—In this paper the Duke de Chaulnes begins with mentioning the authors who have treated on the same subject. Two of the latest of these are Pott and Marg-

 graf, graf, the firſt of whom, we are told, has written a long and very obſcure diſſertation on the fuſible ſalt; and the latter an eſſay, which, in the opinion of our author, is the beſt we have on the ſubject; but at the ſame time he remarks, that it is written very confuſedly, and that ſeveral of the facts related in it are miſrepreſented.

The Duke de Chaulnes obſerves, that almoſt the whole difficulty attending the extraction of this ſalt, is owing to the great quantity of marine ſalt contained in the urine, which chryſtallizes very eaſily, and mixes with the fuſible ſalt. To obviate this, he directs the marine ſalt to be chryſtallized by evaporation, and the fuſible ſalt by cooling. In order to purify the latter, we are adviſed to waſh it firſt with ſome of the cleareſt part of the inſpiſſated liquor, and afterwards with well rectified ſpirit of wine. A ſecond chryſtallization is required, to render the fuſible ſalt perfectly fine and white; and this, we are told, muſt be conducted with great caution, the alkali contained in the ſalt being, in general, ſo volatile as to fly off inſtantly. The manner of conducting this ſecond chryſtallization, tion, with the leaſt poſſible loſs, is very fully deſcribed in the paper itſelf.

On expoſing this ſalt, in a retort, to a ſand heat, the volatile alkali ſoon paſſes into the receiver, and the acid remains in a concrete ſtate. This reſiduum, when heated to a certain degree, vitrifies. Fuſible ſalt that has been chryſtallized only once, yields a white opaque ſubſtance like enamel, which exhales a ſtrong ſmell of muriatic acid. By repeated fuſions it becomes tranſparent, but when expoſed to the air, ſoon deliqueſces and reſumes its opacity; this is attributed to the ſea ſalt it contains. The reſiduum of the ſalt, purified by a ſecond chryſtallization, and expoſed to an intenſe heat, exhibits, when fuſed, the appearance of a beautiful topaz, but on being cooled, becomes perfectly white and tranſparent.

The phoſphoric acid of pure fuſible ſalt, mixed with powdered charcoal, or any other ſubſtance containing phlogiſton, produces phoſphorus.

11. *Experiments for aſcertaining the point of Mercurial Congelation. By Mr.* Thomas Hutchins, *Governor of Albany Fort, in Hudſon's Bay.*—Theſe experiments to determine the freezing point of quickſilver, were made by the direction of the Royal Society, at Albany Fort, in Hudſon's Bay, ſituated in the latitude of 52° 14′ N. and 82° W. longitude from Greenwich.

The inſtruments uſed on this occaſion, were ſimple thermometers, except an apparatus furniſhed by Mr. Cavendiſh, who has deſcribed it in a ſubſequent article, and of which an engraving is given in the work. Of the former, five were mercurial, and the three others ſpirit thermometers; one of the mercurial thermometers was graduated ſo low as 2300° below 0, and the ſcale of one of the ſpirit thermometers deſcended to 160° below the cypher. After deſcribing theſe different thermometers, Mr. Hutchins gives us a table of obſervations from November 23, 1781, till March 24, 1782, made with a view to compare the inſtruments with each other, and to adjuſt, with greater preciſion, the relative degrees on the ſcales.

Next follow accounts of ten experiments: of theſe, the firſt five were made to aſcertain the freezing point of mercury. In all of theſe, the apparatus furniſhed by Mr. Cavendiſh, was uniformly at 40° below 0, when the mercury mercury was frozen. The ſixth and ſeventh experiments were made with a view to aſcertain the greateſt degree of contraction mercury is capable of. The eight and ninth experiments were made with the ſame view as the five firſt, but with a different apparatus, which allowed Mr. Hutchins to have free acceſs to the mercury at all times. In the latter of theſe experiments, half a pound of mercury was frozen in a gallipot; and a mercurial thermometer, which then ſtood at 34°, being put into the part of the quickſilver in the gallipot which was juſt thawed, ſubſided directly to 40°, and became ſtationary. The frozen lump, beaten with a hammer, yielded a dead ſound and flattened, but ſoon crumbled to pieces. The tenth, and laſt experiment, was made on the 26th of January, 1782, and is the moſt intereſting, as it exhibits the curious phenomenon of quickſilver, frozen by the natural cold. It deſerves to be mentioned, that Dr. Black, of Edinburgh, (without knowing what had been done by Mr. Cavendiſh) ſuggeſted to Mr. Hutchins nearly the ſame method as that recommended by Mr. Cavendiſh, for determining the degree of cold at which mercury freezes.

III. *Obſervations on Mr. Hutchins's experiments for determining the degree of cold at which quickſilver freezes. By* Henry Cavendiſh, *Eſq.* F. R. S.—The deſign of this paper is to explain ſome particulars of the apparatus ſent by the author to Mr. Hutchins; and alſo to endeavour to ſhew the cauſe of ſome phenomena which occurred in his experiments; and to point out the conſequences to be drawn from them. The apparatus in queſtion, conſiſted of a ſmall mercurial thermometer, the bulb of which reached about 2½ inches below the ſcale, and was incloſed in a glaſs cylinder, ſwelled at bottom into a ball, which, when uſed, was filled with quickſilver, ſo that the bulb of the thermometer was intirely ſurrounded by it. The exact deſcription Mr. Cavendiſh gives of this apparatus, and of the different thermometers employed at Hudſon's Bay, throw much light on Mr. Hutchins's experiments. The diſagreement which was obſerved in theſe thermometers, was, in Mr. Cavendiſh's opinion, owing ſolely to a faulty manner of adjuſting the boiling point, and to not allowing for the temperature of the air in ſettling the degree of freezing; and as theſe points were examined after they came back, he thinks the experiments ments made with them are juſt as much to be depended on as if they had been truly adjuſted at firſt.

Before his entering into the examination of Mr. Hutchins's experiments, Mr. Cavendiſh has thought it proper to take notice of a phenomenon which occurs in the freezing of water, and which ſhews, that water is capable of being cooled conſiderably below the freezing point, without any congelation taking place; and that, as ſoon as by any means a ſmall part of it is made to freeze, the ice ſpreads rapidly through the remainder of the water. The cauſe of the riſe of the thermometer, when the water begins to freeze, is the circumſtance firſt noticed by Dr. Black and Dr. Irvine, that all, or almoſt all bodies, by changing from a fluid to a ſolid ſtate, or from the ſtate of an elaſtic to that of an inelaſtic fluid, generate heat, or evolve latent heat, as Dr. Black expreſſes it; and that cold is produced by the contrary proceſs. What occurs in the freezing of water, is now known to take place in that of quickſilver, and it occaſioned many remarkable appearances in Mr. Hutchins's experiments. From a review of theſe experiments, Mr. Cavendiſh obſerves, that they all agree agree in ſhewing that the true point at which quickſilver freezes is $38^{\circ}\frac{2}{3}$, or in whole numbers 39° below nothing, it having appeared from the examination of Mr. Hutchins's ſtandard thermometer, after it came home, that 40° thereon, anſwers to $38\frac{2}{3}$ on a thermometer adjuſted in the manner recommended by the Committee of the Royal Society.

After having aſcertained the point of mercurial congelation, Mr. Cavendiſh offers ſome remarks on the contraction of quickſilver in freezing. The very low degrees, he remarks, to which the thermometers were made to ſink in the experiments in queſtion, were owing to this contraction, and not to the cold having been in any degree equal to that ſhewn by the thermometer. In one of Mr. Hutchins's experiments, the thermometer ſunk to 450°, and in another to 448°, though it appeared by the ſpirit thermometers, that in the firſt of theſe inſtances the cold of the mixture was not more than 5° or 6°, and in the latter only $20\frac{1}{2}$ below the point of freezing quickſilver. From theſe, and ſome other ſimilar facts, Mr. Cavendiſh imagines that the contraction which quickſilver ſuffers in freezing, is ſometimes not much leſs than its expanſion by 500° or 510° of heat, that is almoft $\frac{1}{23}$ of its whole bulk.

Mr. Cavendifh concludes this elaborate inquiry with fome obfervations on the cold of freezing mixtures, and here we learn, that by a very ingenious contrivance of diluting fpirit of nitre with fnow, he has, in this country, when the temperature of the air was between 20° and 25°, been able to produce as great a degree of cold within 1°, as was produced in any of the experiments at Hudfon's Bay.

IV. *Hiftory of the Congelation of Quickfilver, By* Charles Blagden, M.D. F.R.S. *Phyfician to the Army.*—The experiments at Hudfon's Bay have determined a point upon which philofophers not only were much divided in their opinion, but alfo entertained, in general, very erroneous fentiments; and in the general progrefs of fcience, it is always ufeful at intervals, and efpecially when any confiderable advance has been made, to look round and contemplate the profpect left behind. Thus our actual fituation is more diftinctly comprehended, and a better judgement may be formed of what remains to be done. Thefe confiderations have induced Dr. Blagden to communicate this account of the different obſervations and experiments he has been able to collect relative to the congelation of quickſilver. Many of theſe are recorded in books not eaſily procured, and in languages little underſtood by the learned of this country; but theſe are not the only circumſtances that will recommend the paper now before us to the notice of philoſophical readers, the value of the materials of which it is compoſed being greatly enhanced by the judicious manner in which they are arranged, and by the great number of intereſting remarks with which the work is interſperſed.

In an introduction to his work, Dr. Blagden obſerves, that although "many obvious "circumſtances rendered it improbable, that "the term of mercurial congelation ſhould be "five or ſix hundred degrees below 0 of Fahren- "heit's ſcale, as had been at firſt ſuppoſed; "yet ſcarcely any one ventured to imagine "that it was ſhort of 100°." Mr. Hutchins, however, continues our author, "has "clearly proved, that even this number is far "beyond the truth; and that quickſilver "freezes in a degree of cold not exceeding "that which ſometimes occurs in the northern

"parts

" parts of Europe, and frequently in the more " rigorous climates of Aſia and America. It " now appears that quickſilver, ſo far from " containing any eſſential principle of fluidity, " does not differ from ſome of the other " metals, in its melting point, nearly ſo much " as they differ among themſelves; and as it " is malleable in its ſolid ſtate, and after its " calcination recovers its metallic form by " heat alone, without the addition of inflam-" mable matter, there can be no doubt but " it muſt be ranked among the perfect " metals."

The work itſelf is divided into two parts. In the firſt, the author deſcribes the various attempts which have been made to render quickſilver ſolid by frigorific mixtures, beginning with Profeſſor Braun's experiment in 1759, which he conſiders as the firſt that eſtabliſhed the fact that mercury can be made ſolid by a diminution of its heat. In the ſecond part, Dr. Blagden enumerates the many inſtances in which that effect has been produced by natural cold. In a few of theſe inſtances, he obſerves, " the effect was ſo palpable and ob-" vious, as to ſtrike with immediate convic-" tion; but in moſt it has never been even

" suspected, till the present time; the strange " appearances which often occurred, being " imputed to any other rather than the real " cause, though they are now found to carry " with them a force of internal evidence " which establishes the truth beyond all " doubt." Many of the contradictory appearances which had perplexed former observers, now become explicable by the determination of the point at which mercury congeals, by its adhesion to other bodies on becoming solid, by its contraction in consequence of its being congealed, and by the degree of cold it is capable of receiving beyond its freezing point before it actually congeals. In the course of the inquiry, Dr. Blagden has not omitted to avail himself of the doctrine of latent heat, in accounting for several phenomena which would otherwise be inexplicable.

Among the instruments sent to Hudson's Bay, were two spirit thermometers. These were sent at the request of our author, and his intention in recommending them, it seems, was to discover what degree of cold the freezing mixture produced; and to obtain a more exact comparison of the relative contractions of mercury and alcohol, by making their simul-

ſimultaneous deſcents on a more extended ſcale, or as long as both of them ſhould continue to contract regularly. He does not think it proper, in the preſent inquiry, to enter into any detail of the obſervations that were made to ſettle the relative contractions of quickſilver and ſpirits by means of theſe inſtruments, but contents himſelf with mentioning, that on one of them, the 29th degree below 0, and on the other the 30th, were found to correſpond with 40° of the ſmall mercurial thermometers, or more preciſely with the point that would have been 39° upon an exact ſtandard inſtrument. By theſe inſtruments, the author obſerves, it is now determined, that the greateſt effect of a mixture of ſnow and ſmoaking acid of nitre, even with the advantage of ſuch natural cold as congealed the quickſilver expoſed to it, was only to diminiſh the heat to ſuch a degree as would correſpond with 45° or 46° of a ſtandard mercurial thermometer; and as this was likewiſe the greateſt degree of cold indicated during the moſt intenſe natural cold of Hudſon's Bay, according to the obſervations made by Mr. Hutchins, during a ſeries of years, our author thinks we are perhaps authoriſed to to conclude, that 46 degrees below o, is the extreme both of natural and artificial cold.

Although the preference due to ſpirit thermometers, in experiments where degrees of cold below the freezing point of mercury are to be determined, is clearly eſtabliſhed by Mr. Hutchins's obſervations; yet our author very judiciouſly obſerves, that from the boiling point to 39° or 40° below o, the uſe of quickſilver for thermometers muſt be conſidered as unexceptionable, all ſuſpicion of its irregular contraction within thoſe bounds being removed, by ſuch a complete explanation of the cauſe upon which its anomalous deſcent in the lower part of the ſcale depends. On this principle there might, he thinks, be ſome propriety in conſtructing thermometers of mercury, to fix the cypher at its point of congelation, and thence reckon the degrees of heat upwards.

v. *Experiments relating to Phlogiſton, and the ſeeming Converſion of Water into Air. By* Joſeph Prieſtley, L.L.D. F.R.S. — There are few ſubjects that have occaſioned more perplexity to chymiſts than that of phlogiſton. It was the great diſcovery of Stahl, that this principle is transferable from one ſubſtance to to another, how different foever in their other properties, and therefore is the fame thing in them all. But an idea that this principle could not be exhibited in a feparate form, has given an air of myftery to the fubject. Of late, M. Lavoifier and others have contended, that the whole doctrine of phlogifton has been founded on miftake, and that in all cafes in which it was thought that bodies parted with phlogifton, they, inftead of lofing, acquired fomething, and, in general, an addition of fome kind of air. The experiments on mercury by M. Lavoifier were fo favourable to this opinion, that our author acknowledges he was himfelf much inclined to adopt it. His friend, Mr. Kirwan, indeed, always held that phlogifton was the fame thing with inflammable air, and he has fufficiently proved this, we are told, from many experiments and obfervations made by our author and others. Dr. Prieftley did not, however, accede to it, he informs us, till he difcovered it by direct experiments, made with general and indeterminate views, in order to afcertain fomething concerning a fubject which had given himfelf and others fo much trouble.

He began with repeating ſome experiments in which he had found that inflammable air made red hot in flint-glaſs tubes, gave them a black tinge, and was in a great meaſure abſorbed, which he found to be owing to the calx of lead in the glaſs attracting phlogiſton from the inflammable air. After this, he threw the focus of a burning lens upon a quantity of minium in a large receiver filled with inflammable air, confined by water, and the reſult was, that the metal became firſt black, and then run in the form of perfect lead, the air, at the ſame time, diminiſhing at a great rate. He afterwards expelled from a quantity of minium all the phlogiſton, by giving it a red heat when mixed with ſpirit of nitre, and immediately uſing it as in the preceding experiment, he reduced 101 ounce meaſures of inflammable air to two. Theſe experiments convinced him, that the inflammable air went totally, and without decompoſition, into the lead. He now tried all the other kinds of air in the ſame manner, but in none of them did he procure any thing from the minium beſides glaſs of lead, except in alkaline air, and vitriolic acid air. In the latter of theſe only a ſmall quantity of lead was procured; but in alkaline air, we we are told, lead ſeems to be formed from the minium as readily as in inflammable air. By taking the electric ſpark in alkaline air, he has repeatedly converted it into three times as much pure inflammable air; hence he thinks it probable, that one of them is ſome modification of the other, or a combination of ſomething elſe with the other: and ſome experiments which the author relates prove that alkaline air is the compound, and inflammable air the more ſimple ſubſtance of the two.

Having thus produced lead in inflammable air, Dr. Prieſtley attempted to revive other metals from their calces by the ſame means, and he ſucceeded very well with tin, biſmuth, and ſilver; tolerably well with copper, iron, and regulus of cobalt; but not at all with regulus of antimony, regulus of arſenic, zinc, or the metal of manganeſe. He was deſirous alſo to aſcertain the quantity of phlogiſton that enters into the compoſition of the ſeveral metals; he found more difficulty in this than he had expected, as it was not eaſy to revive the whole of any quantity of calx completely, but after many trials, he thinks he may venture to ſay, that

| | | | | |
|---|---|---|---|---|
| An ounce of | lead | abſorbs | 100 | ounce meaſures of inflammable air. |
| ——— | tin | ——— | 377 | ——— |
| ——— | biſmuth | ——— | 185 | ——— |
| ——— | iron | ——— | 890 | ——— |

The abſorption, *in toto*, of inflammable air, without decompoſition, by the different calces in theſe experiments, Dr. Prieſtley conſiders as a proof that phlogiſton is the ſame thing with this air, and that it is contained in a combined ſtate in metals, juſt as fixed air is contained in calcareous ſubſtances.

Beſides the formation of metals from their calces, our author has had other proofs of inflammable air containing phlogiſton; having, by means of it, been able to make phoſphorus, nitrous air, liver of ſulphur, and ſulphur itſelf. Thus, by throwing the focus of the lens on a quantity of that glaſſy matter, which is made from calcined bones by oil of vitriol in inflammable air, phoſphorus was formed; but this experiment, as we learn, ſucceeded much better, when alkaline air was employed inſtead of inflammable air.

Doctor Prieſtley produces nitrous air, by uniting its two conſtituent principles, nitrous vapour, and inflammable air. The experiments which he deſcribes as the moſt unexceptionable for this purpoſe, conſiſted in expoſing what what he calls a *nitrated calx* of lead (formed by uniting nitrous vapour with minium), in a receiver filled with inflammable air, to the focus of the lens. In this procefs there was a diminution of two-thirds of the inflammable air, and lead was revived from the calx; what remained of the air was ftrongly nitrous, and this, our author fuppofes, muft have been formed by the nitrous vapour contained in the calx, and the inflammable air in the receiver. —He procured liver of fulphur, by throwing the focus of the lens upon vitriolated tartar in inflammable air; and to produce fulphur, he threw the focus of the lens on oil of vitriol, contained in a hollow earthen veffel, and evaporated it to drynefs in a receiver, filled with inflammable air, in confequence of which the infide of the receiver acquired a whitifh incruftation, which proved to be fulphur.

Charcoal has generally been fuppofed to be indeftructible, except by a red heat in contact with air, but our author finds that it is perfectly deftructible in *vacuo*, and by the heat of a burning lens, almoft wholly convertible into inflammable air; fo that nothing remains befides a fmall quantity of white afhes, fo fmall indeed, that the quantity produced from many pounds

 of of wood cannot be ſuppoſed to weigh a grain, Dr. Prieſtley ſhews, that the great weight of aſhes produced by burning wood in the open air, ariſes from what is attracted by them from the air. When the charcoal is perfectly well made, the air, which he gets in this manner, is wholly inflammable air, without any fixed air in it. He adds, that wood or charcoal is even reſolvable into inflammable air, in a good earthen retort, and a fire that would about melt iron.

The learned author next mentions ſome experiments, which prove the generation of fixed air from dephlogiſticated and inflammable airs, and confirm the opinion of Mr. Kirwan, that fixed air is a factitious ſubſtance compoſed of dephlogiſticated air and phlogiſton. From ſome of theſe experiments it appeared that three ounce meaſures of dephlogiſticated air go into the compoſition of two ounce meaſures of fixed air.

The experiments relating to phlogiſton are followed by others, concerning the ſeeming converſion of water into air. This ſeeming converſion took place, when water mixed with calcareous earth, was expoſed in an earthen retort to a red heat. In this proceſs nothing came came over in the form of ſteam, but there was a great quantity of air, ſeveral hundred times more than the bulk of the water, and which, when the fixed air that was in it was extracted from it, was ſuch as a candle would juſt burn in. Our author was much diſconcerted on finding, that when the experiment was made in a coated glaſs retort, the water came over in the form of ſteam, and little or no air was produced. His ſpeculations on the ſubject were various, but at that time altogether ineffectual.

Being ſatisfied, that the production of air depended very much upon the retort itſelf, he uſed it only with water, without any lime or earthy ſubſtance. In all his trials in this way, however, he obſerved, that very little of this air was procured, till all the water that could be poured out of the retort was evaporated, for the difference in the produce was very little, whether he expoſed the retort to the fire quite full, or with only an ounce meaſure of water in it.

The limits of our journal will not allow us to enter into an account of all the experiments which the learned author made to ſatisfy his own doubts, or to obviate the objections of his friends friends on the ſubject; we ſhall, therefore, proceed to that part of his paper where the myſtery is explained.—Having obſerved, that the purity of the air which he procured, depended upon the ſtate of that which was contiguous to the retort, and that ſome communication with the atmoſphere was neceſſary to the production of any air; he was deſirous of aſcertaining what the influence of the external air in this caſe really was. For this purpoſe, he incloſed an earthen retort filled with moiſtened clay in a large glaſs receiver open at both ends, through the upper orifice of which he thruſt the neck of the retort, luting it ſo as to be perfectly air tight; and placing the receiver in a baſon of water, by which the air within was cut off from all communication with the external air; he fitted to the mouth of the retort a glaſs tube, through which he could receive whatever was produced in the proceſs. In this ſituation, he heated the retort, by means of Mr. Parker's excellent burning lens (the ſame that had been uſed in the former experiments on phlogiſton) and found that air was received, through the tube communicating with the inſide of the retort, as uſual; but, at the ſame time, the water roſe within the receiver. This effect

 might might be owing to a phlogiſtication of the air within the receiver; but it was ſoon diminiſhed far beyond the utmoſt limit of that proceſs, ſo that very little of it remained; and examining this air, the author found it to be but very little worſe than atmoſpheric air, as that which came from the retort was a little better.

This experiment made it probable, that the air on the outſide of the receiver had actually paſſed through it, only a little purified in its paſſage; and yet it was contrary to all the known principles of hydroſtatics, and to any thing known in chemiſtry, that air ſhould be tranſmitted through a veſſel of this kind, and in a direction contrary to that in which it would have been forced by the preſſure of the atmoſphere; while the water with which the clay was moiſtened went the other way. This, however, appeared to be the caſe by other deciſive experiments related by the author, and from which he infers, that the clay of the earthen retort being thus heated, deſtroys, for a time, the aerial form of whatever is expoſed to the outſide of it, which aerial form it recovers, after it has been tranſmitted in combination from one part of the clay to another, till it has reached the inſide of the retort, while the water water is drawn through it in the contrary direction.

Among the other papers in this volume are *some experiments upon the ochra friabilis nigro fusca of Da Costa, Hist. Foss. p.* 102; *and called by the miners of Derbyshire, black wadd, by* Josiah Wedgewood, *F. R. S.*; *and a description of an improved air pump, by Mr.* Tiberius Cavallo, *F. R. S.*

